# Safety and feasibility of aortic crossclamp for thrombosed acute type A aortic dissection

**DOI:** 10.1016/j.xjon.2025.10.026

**Published:** 2025-11-10

**Authors:** Yoshinori Nakahara, Akira Marui, Yuito Okada, Kohei Sumi, Ryogen Yun, Makoto Ono, Tomohiro Iwakura

**Affiliations:** Department of Cardiovascular Surgery, Sakakibara Heart Institute, Tokyo, Japan

**Keywords:** aortic dissection, surgery, crossclamp, thrombosis, false lumen, stroke

## Abstract

**Objective:**

Aortic crossclamping in acute type A aortic dissection surgery facilitates proximal procedures during systemic cooling and reduces cardiopulmonary bypass time. However, concerns exist about embolization risk when crossclamping patients with thrombosed false lumen. We compared safety and feasibility of ascending aortic crossclamping between thrombosed and patent false lumen in acute type A aortic dissection.

**Methods:**

This single-center retrospective study analyzed 1550 consecutive acute type A aortic dissection patients undergoing surgery between January 2004 and December 2023. Of these, 1407 patients completed aortic crossclamping and were divided into thrombosed false lumen (400 patients [28.4%]) and patent false lumen (1007 patients [71.6%]) groups. Propensity score matching yielded 400 matched pairs.

**Results:**

After propensity score matching, both groups showed similar preoperative characteristics. No significant differences existed in new onset ischemic stroke (5.8% vs 5.5%; *P* > .99) or spinal cord injury (1.3% vs 1.0%; *P* > .99). Thirty-day mortality was lower in the thrombosed false lumen group (2.3% vs 5.3%; *P* = .04). Sensitivity analyses adjusting for residual imbalances, including salvage surgery and redo surgery, confirmed consistent stroke safety (*P* = .64) and showed attenuated but directionally consistent mortality findings. Multivariate logistic regression revealed that a thrombosed false lumen was not an independent risk factor for new ischemic stroke (odds ratio, 1.04; 95% CI, 0.61-1.73; *P* = .88), but was protective for 30-day mortality (odds ratio, 0.47; 95% CI, 0.21-0.95; *P* = .047).

**Conclusions:**

Ascending aortic crossclamping was safe and feasible in acute type A aortic dissection surgery regardless of false lumen status. Thrombosed false lumen presence does not increase neurological complications or early mortality risk.

## Graphical Abstract

**Figure:**
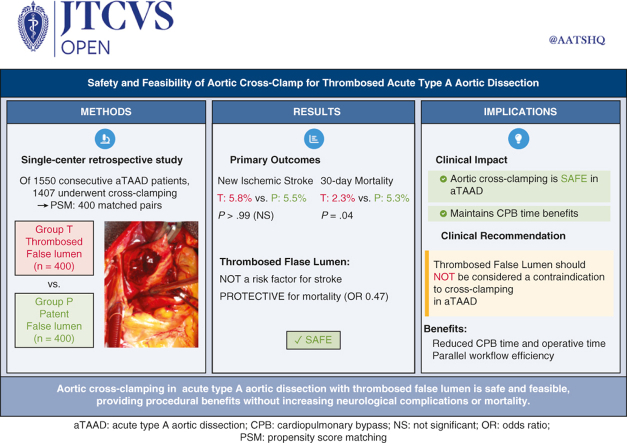

**Figure:**
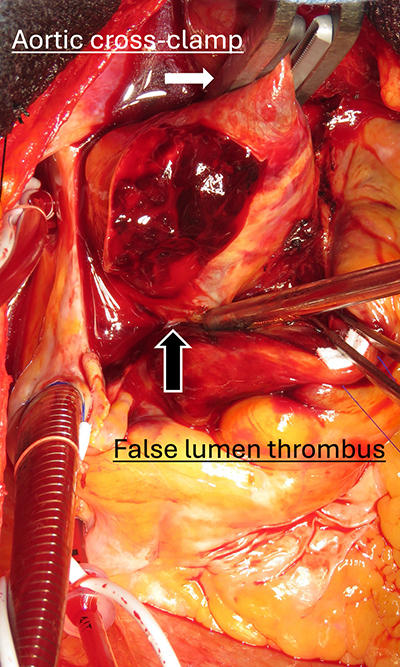
Crossclamp applied to ascending aorta with thrombosed false lumen during aTAAD surgery.

**Central Message**

Aortic crossclamping in acute type A aortic dissection with thrombosed false lumen is safe and feasible, showing no increased risk of stroke or mortality compared with patent false lumen cases.

**Perspective**

Despite theoretical concerns about embolization, this large propensity score-matched study demonstrates that aortic crossclamping can be safely performed in patients with acute type A aortic dissection with thrombosed false lumen, without increasing neurological complications or early mortality.

Acute type A aortic dissection (aTAAD) remains 1 of the most challenging cardiovascular emergencies, with surgical mortality rates ranging from 9% to 11% in Japanese nationwide databases to 17% to 19% in Western registries.^1^^,^^2^ Surgical management typically involves replacement of the affected ascending aorta under cardiopulmonary bypass (CPB) with hypothermia and circulatory arrest.^3^^,^^4^

Aortic crossclamping during aTAAD surgery offers several potential advantages. It facilitates proximal procedures during the cooling phase of CPB, allowing for a parallel workflow, and reducing overall operative times. Additionally, it limits the duration of CPB and operation, which has been associated with reduced morbidity and improved outcomes.^5^^,^^6^ However, there are theoretical concerns regarding the application of crossclamps in patients with aTAAD, especially those with a thrombosed false lumen. Specifically, there is a risk of dislodging thrombus material and causing downstream embolization, particularly cerebral embolism.^7^^,^^8^

The optimal approach for patients with thrombosed false lumen remains debated.^9^^,^^10^ Some surgeons avoid crossclamping due to embolic concerns, preferring deep hypothermic circulatory arrest, which may extend CPB duration.^11^^,^^12^ At our institution, we routinely perform aortic crossclamping regardless of false lumen thrombosis status. We evaluated whether aortic crossclamping in aTAAD with thrombosed false lumen increases neurological complications or mortality compared with patent false lumen cases.

## Methods

### Study Design and Population

This single-center, retrospective study included consecutive patients who underwent emergency surgery for aTAAD at our institution between January 2004 and December 2023. This study was approved by the Ethical Committee of Sakakibara Heart Institute on May 9, 2024 (approval No.: 25-002), and individual patient consent was waived due to the retrospective nature of the study.

From a total of 1550 patients, 1407 (90.8%) underwent aortic crossclamping and were included. Major reasons for avoiding crossclamp included arterial cannulation from the left ventricular apex (n = 60 [43%]), severe adhesion in redo surgery (n = 13 [9%]), and concomitant on-pump beating coronary artery bypass grafting (n = 7 [5%]). Three cases initially required declamping but were included in the final analysis. Of these, 1 patient developed hypotension in both upper limbs, another experienced hypotension in the left upper limb, and the third experienced aortic rupture due to cannulation failure. Patients were divided based on false lumen status: thrombosed false lumen (Group T) (n = 400 [28.4%]) and patent false lumen (Group P) (n = 1007 [71.6%]). Thrombosed false lumen included both complete thrombosis (n = 328 [82.0%]) and partial thrombosis (n = 72 [18.0%]) of the false lumen in the ascending aorta.

All surgeries were classified according to their emergency or urgent status. Salvage surgery was defined as emergency surgery performed in patients with cardiac arrest or extreme hemodynamic instability requiring cardiopulmonary resuscitation and/or mechanical circulatory support (eg, extracorporeal membrane oxygenation).

### Surgical Technique

All operations were performed through a median sternotomy under general anesthesia. Aortic crossclamping was avoided in patients with significant ascending aortic dilatation (>50-60 mm) due to rupture risk. Arterial cannulation site selection was based on preoperative imaging and clinical assessment. Femoral artery cannulation was avoided when there was a concern for retrograde embolism from significant atherosclerotic disease or for intraoperative malperfusion due to dissection extending to the cervical branches. Alternative cannulation sites, including the subclavian artery, left ventricular apex, or ascending aorta, were selected based on these clinical considerations and surgeon's preference.

Our surgical decision-making algorithm for extended arch replacement was based on the following criteria: entry-oriented surgery as the primary principle, aggressive arch replacement in young patients with a patent false lumen and the presence of arch dilatation. The application of this algorithm resulted in a total arch replacement rate of 34.4% (484 out of 1407).

**Figure 1:**
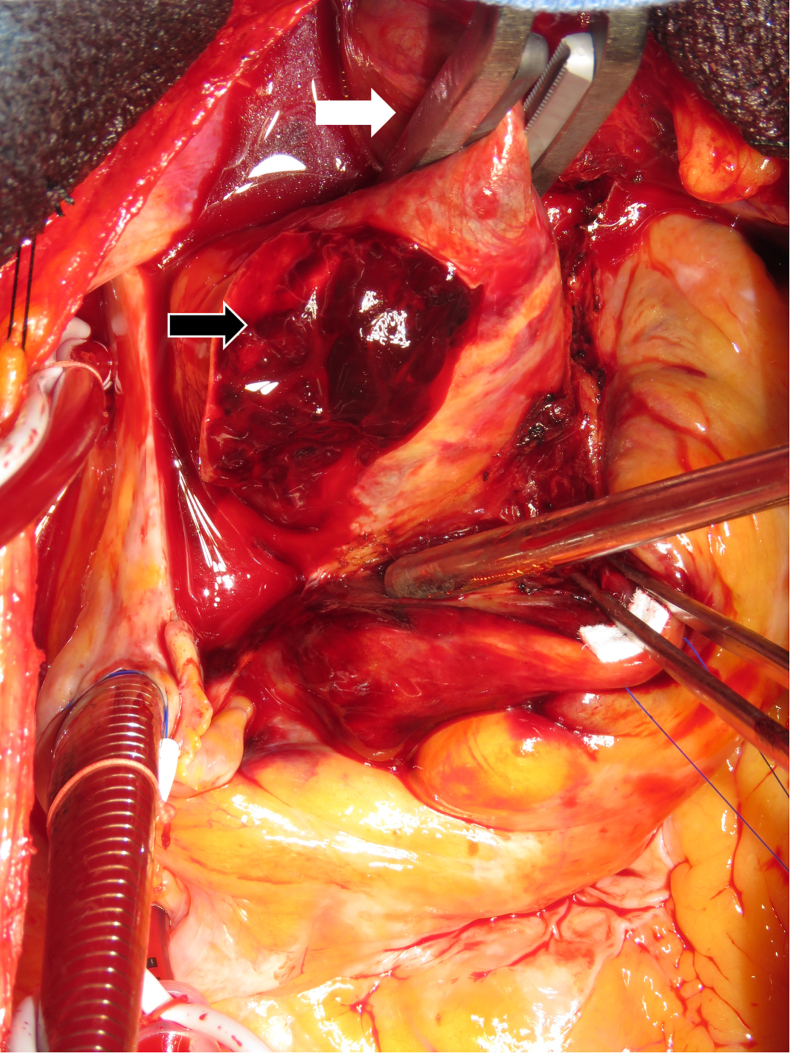
Intraoperative image of aortic crossclamping in thrombosed false lumen. Surgical technique demonstrating crossclamp application to the ascending aorta proximal to the innominate artery in acute type A aortic dissection with thrombosed false lumen. The clamp is positioned to allow safe proximal procedures during the cooling phase of cardiopulmonary bypass. *White arrow* indicates aortic crossclamp; *black arrow* shows fresh thrombus in the false lumen exposed through adventitial incision.

After establishment of CPB, systemic cooling was initiated, and the ascending aorta was crossclamped proximal to the innominate artery (Figure 1). Proximal procedures, including reinforcement of the aortic wall, aortic valve repair or replacement, and root surgery were performed during the cooling phase. Our routine proximal reinforcement includes false lumen thrombus removal, BioGlue (CryoLife) application, and felt reinforcement of inner and outer felt strips with *U*-sutures, typically requiring approximately 20 minutes. Once the target temperature was reached (24 °C-25 °C), circulatory arrest was established and the distal anastomosis was performed. This technique is demonstrated in Video 1.

### Data Collection and Outcome Measures

Baseline patient characteristics, operative variables, and postoperative outcomes were collected from our institutional database and electronic medical records. The primary outcomes were new-onset ischemic stroke and 30-day all-cause mortality. New-onset ischemic stroke was defined as symptomatic stroke or brain infarction identified by any imaging modality occurring intraoperatively or postoperatively. Patients with preoperative neurological symptoms due to malperfusion were excluded from the new-onset stroke outcome to isolate procedural cerebral ischemic events. Secondary outcomes included spinal cord injury, acute kidney injury (defined as Kidney Disease Improving Global Outcomes stage 2 or greater), requirement for renal replacement therapy, respiratory failure requiring prolonged ventilation (>72 hours), or tracheotomy.

### Statistical Analysis

Statistical analysis was reviewed and verified by a biostatistician and coauthor (Y.O.). To mitigate selection bias and baseline differences between groups, propensity score matching was performed using the following prespecified covariates based on clinical expertise and literature review: age, sex, body surface area, hypertension, dyslipidemia, diabetes mellitus, preoperative renal failure (defined by a creatinine level >2 mg/dL), preoperative malperfusion, shock status, neurological symptoms, arterial cannulation site, total arch replacement, and root surgery.

Patients were matched 1:1 using a greedy matching algorithm with a caliper width of 0.2 SDs of the logit of the propensity score, without replacement. The quality of matching was assessed by calculating standardized mean differences (SMDs), with values < 0.1 considered indicative of good balance. Missing data were handled using complete case analysis.

To assess the robustness of our findings and address potential confounding by residual imbalances after primary matching (particularly salvage surgery with SMD = 0.132 and redo surgery with SMD = 0.181), we performed 2 complementary sensitivity analyses.

• Enhanced propensity score matching: We repeated propensity score matching with salvage surgery and redo surgery included as additional matching variables in addition to all variables used in the primary analysis. This enhanced matching used the same matching algorithm and caliper width as the primary analysis.
• Multivariable regression sensitivity analysis: We performed multivariable logistic regression for 30-day mortality with redo surgery added as an additional covariate to the primary regression model to assess potential confounding effects of this variable. Continuous variables are presented as mean ± SD or median with interquartile range, as appropriate, and were compared using the Student *t* test or Mann-Whitney *U* test. Categorical variables are presented as frequencies and percentages and were compared using χ^2^ or Fisher exact test. Multivariate logistic regression analysis was performed to identify independent risk factors for new-onset ischemic stroke and 30-day mortality. Covariates were selected based on clinical expertise and literature review. For the new-onset ischemic stroke analysis, the preoperative malperfusion variable was excluded from the regression model to avoid perfect separation because patients with neurological malperfusion were excluded from the new-onset stroke definition by design, resulting in 0 variance for this outcome. Statistical analyses were conducted using R software version 4.4.3 (R Foundation for Statistical Computing).

## Results

### Baseline Characteristics

After matching, 400 patients in each group were successfully matched, resulting in well-balanced baseline characteristics (Table 1). Although most covariates achieved good balance (SMD <0.1), residual imbalances remained for salvage surgery, redo surgery, Marfan syndrome, and partial arch replacement.

**Table 1:** Baseline patient characteristics and operative details before and after propensity score matching

Variable	Before matching			After matching		
	Group T (n = 400)	Group P (n = 1007)	SMD∗	Group T (n = 400)	Group P (n = 400)	SMD∗
Patient demographic						
Age (y)†	72 (65-80)	67 (56-75)	0.454	72 (65- 80)	73 (64- 80)	<0.001
Female sex†	232 (58.0)	464 (46.1)	0.240	232 (58.0)	226 (56.5)	0.030
Body surface area (m^2^)†	1.6 (1.5-1.7)	1.7 (1.5-1.8)	0.270	1.6 (1.5-1.7)	1.6 (1.4-1.8)	0.018
Body mass index	23.0 [21.2, 25.8]	23.5 [21.0, 26.0]	0.074	23.0 [21.2, 25.8]	23.1 [20.8, 25.6]	0.031
Comorbidities						
Hypertension†	296 (74.0)	741 (73.6)	0.009	296 (74.0)	291 (72.8)	0.028
Dyslipidemia†	98 (24.5)	200 (19.9)	0.112	98 (24.5)	89 (22.2)	0.053
Diabetes mellitus†	26 (6.5)	49 (4.9)	0.071	26 (6.5)	26 (6.5)	<0.001
Creatinine >2 mg/dL†	12 (3.0)	50 (5.0)	0.101	12 (3.0)	13 (3.2)	0.014
Marfan syndrome	2 (0.5)	26 (2.6)	0.170	2 (0.5)	1 (0.3)	0.410
Coronary artery disease	23 (5.8)	49 (4.9)	0.039	23 (5.8)	26 (6.5)	0.031
Clinical presentation						
Malperfusion†	33 (8.2)	229 (22.7)	0.409	33 (8.2)	33 (8.2)	<0.001
Shock†	78 (19.5)	177 (17.6)	0.050	78 (19.5)	83 (20.8)	0.031
Neurological symptoms†	20 (5.0)	102 (10.1)	0.195	20 (5.0)	18 (4.5)	0.024
Paralysis	12 (3.0)	71 (7.1)	0.186	12 (3.0)	13 (3.2)	0.014
Rupture	89 (22.2)	161 (16.0)	0.160	89 (22.2)	92 (23.0)	0.018
Salvage surgery	10 (2.5)	43 (4.3)	0.098	10 (2.5)	20 (5.0)	0.132
Operative variables						
Femoral artery perfusion†	314 (78.5)	841 (83.5)	0.128	314 (78.5)	326 (81.5)	0.075
Subclavian artery perfusion	48 (12.0)	88 (8.7)	0.107	48 (12.0)	36 (9.0)	0.098
Ascending aorta perfusion	38 (9.5)	78 (7.7)	0.063	38 (9.5)	38 (9.5)	<0.001
Apex perfusion	0 (0.0)	0 (0.0)	–	0 (0.0)	0 (0.0)	–
Ascending aortic replacement	329 (82.2)	582 (57.8)	0.554	329 (82.2)	328 (82.0)	0.007
Partial arch replacement	1 (0.2)	11 (1.1)	0.103	1 (0.2)	7 (1.8)	0.151
Total arch replacement†	70 (17.5)	414 (41.1)	0.537	70 (17.5)	65 (16.2)	0.033
Root surgery†,‡	18 (4.5)	145 (14.4)	0.343	18 (4.5)	21 (5.2)	0.035
Redo surgery	4 (1.0)	30 (3.0)	0.142	4 (1.0)	15 (3.8)	0.181
Coronary artery bypass grafting	10 (2.5)	81 (8.0)	0.250	10 (2.5)	15 (3.8)	0.072
Aortic valve replacement/repair	26 (6.5)	176 (17.5)	0.343	26 (6.5)	34 (8.5)	0.076

Values are presented as n (%) for categorical variables and median (interquartile range) for continuous variables. *Group T*, Thrombosed false lumen; *Group P*, patent false lumen; *SMD*, standardized mean difference.

∗ SMD absolute values ≤ 0.10 indicate negligible differences (excellent balance), 0.10 to 0.25 indicate acceptable differences, and >0.25 indicate substantial imbalances. Residual imbalances with SMD >0.1 (specifically salvage surgery [0.132], redo surgery [0.181], Marfan syndrome [0.410], and partial arch replacement [0.151]) were addressed through comprehensive sensitivity analyses, which included enhanced propensity score matching for salvage surgery and redo surgery (Tables E1 and E2).

† Variables included in the propensity score-matching model.

‡ Root surgery includes Bentall procedure or valve-sparing surgery.

### Anatomical and Morphological Characteristics on Computed Tomography

The preoperative anatomical and morphological characteristics were summarized in Table 2. Dissection localized to the ascending aorta (DeBakey type II) was significantly more common in Group T than in Group P (23.2% vs 13.5%; *P* < .001). The distribution of the primary entry location also differed significantly. An entry tear in the ascending aorta (51.5% vs 61.4%; *P* < .001) and aortic arch (15.5% vs 27.5%; *P* < .001) were both significantly less frequent in Group T compared with Group P. Conversely, cases with an unknown entry location were significantly more prevalent in Group T (30.5% vs 8.2%; *P* < .001).

**Table 2:** Anatomical and morphological characteristics on computed tomography

Variable	Before matching			After matching		
	Group T (n = 400)	Group P (n = 1007)	*P* value	Group T (n = 400)	Group P (n = 400)	*P* value
Dissection type						
Ascending localized (DeBakey type II)	93 (23.2)	136 (13.5)	<.001	93 (23.2)	85 (21.2)	.55
Patent type	0 (0.0)	1007 (100.0)	<.001	0 (0.0)	400 (100.0)	<.001
Thrombosed type	328 (82.0)	0 (0.0)	<.001	328 (82.0)	0 (0.0)	<.001
Partial thrombosed type	72 (18.0)	0 (0.0)	<.001	72 (18.0)	0 (0.0)	<.001
Primary entry location						
Aortic root	5 (1.2)	11 (1.1)	>.99	5 (1.2)	1 (0.2)	.22
Ascending aorta	206 (51.5)	618 (61.4)	<.001	206 (51.5)	293 (73.2)	<.001
Aortic arch	62 (15.5)	277 (27.5)	<.001	62 (15.5)	69 (17.2)	.57
Descending aorta	5 (1.2)	18 (1.8)	.63	5 (1.2)	9 (2.2)	.42
Unknown	122 (30.5)	83 (8.2)	<.001	122 (30.5)	28 (7.0)	<.001

Values are presented as n (%). *Group T*, Thrombosed false lumen; *Group P*, patent false lumen.

The aortic features in the three cases requiring declamping were as follows. One case of rupture involved an ascending aorta dilated to 48 mm and was of the thrombosed false lumen. The 2 cases presenting with upper extremity hypotension were both from the patent false lumen group, with the entry located in the ascending aorta.

### Operative Variables and Early Outcomes

Operative variables after matching are presented in Table 1, and early outcomes are presented in Table 3. After matching, there were no significant differences in CPB time (109 vs 113 minutes; *P* = .21) or crossclamp time (78 vs 78 minutes; *P* = .50). Circulatory arrest time was slightly longer in Group T (24 vs 23 minutes; *P* = .03), whereas total operation time was shorter (200 vs 208 minutes; *P* = .013). No significant differences existed in new ischemic stroke (5.8% vs 5.5%; *P* > .99) or spinal cord injury (1.3% vs 1.0%; *P* > .99). Thirty-day mortality was significantly lower in Group T (2.3% vs 5.3%; *P* = .04). Acute kidney injury was lower in Group T (7.8% vs 14.2%; *P* = .005), but the difference in requirement for renal replacement therapy was not significant (2.3% vs 4.5%; *P* = .12).

**Table 3:** Comparison of early outcomes before and after propensity score matching

Outcome	Before matching			After matching		
	Group T (n = 400)	Group P (n = 1007)	*P* value	Group T (n = 400)	Group P (n = 400)	*P* value
Operative time						
Cardiopulmonary bypass time (min)	109 (98-131)	130 (106-176)	<.001	109 (98-131)	113 [98-136]	.21
Aortic crossclamp time (min)	78 (67-94)	95 (73-136)	<.001	78 (67-94)	78 (66-98)	.50
Circulatory arrest time (min)	24 (20-29)	26 (20-37)	<.001	24 (20-29)	23 (19-28)	.03
Operation time (min)	200 (172-243)	236 (190-300)	<.001	200 (172-243)	208 (180-255)	.01
Early outcomes						
New ischemic stroke	23 (5.8)	62 (6.2)	.87	23 (5.8)	22 (5.5)	>.99
Spinal cord injury	5 (1.3)	17 (1.7)	.77	5 (1.3)	4 (1.0)	>.99
Acute kidney injury∗	31 (7.8)	178 (17.7)	<.001	31 (7.8)	57 (14.2)	.005
Requirement for renal replacement therapy	9 (2.3)	73 (7.2)	<.001	9 (2.3)	18 (4.5)	.12
Respiratory failure†	28 (7.0)	115 (11.6)	.02	28 (7.0)	39 (9.8)	.19
30-d mortality	9 (2.3)	66 (6.6)	.002	9 (2.3)	21 (5.3)	.04

Values are presented as n (%) for categorical variables and median (interquartile range) for continuous variables. Sensitivity analyses with enhanced propensity score matching (salvage surgery and redo surgery included as matching variables) and multivariable regression with additional covariates showed consistent results (Table E2 and Table 4). *Group T*, Thrombosed false lumen; *Group P*, patent false lumen.

∗ Acute kidney injury is defined as Kidney Disease Improving Global Outcomes stage 2 or greater.

† Respiratory failure is defined as prolonged ventilation >72 hours or requirement for tracheotomy.

### Sensitivity Analyses

To address residual imbalances after primary matching, particularly salvage surgery and redo surgery, we performed 2 sensitivity analyses. Both sensitivity analyses confirmed consistent stroke safety (5.8% vs 5.1%; *P* = .64 in enhanced matching; odds ratio [OR], 1.04; *P* = .88 in regression). The mortality benefit observed in primary analysis was attenuated in both sensitivity analyses (2.3% vs 4.3%; *P* = .123 in enhanced matching; OR, 0.48; 95% CI, 0.21-0.97; *P* = .054 in regression with redo surgery adjustment), although the direction of protective effect remained consistent (Tables E1 and E2 and Table 4).

**Table 4:** Risk factors for 30-day mortality: Logistic regression analysis

Variable	Primary analysis			Sensitivity analysis		
	Odds ratio	95% CI	*P* value	Odds ratio	95% CI	*P* value
Malperfusion	3.77	2.22-6.39	<.001	3.75	2.21-6.37	<.001
Salvage surgery	5.36	2.50-11.60	<.001	5.43	2.53-11.76	<.001
Shock	2.71	1.45-4.93	.001	2.71	1.45-4.94	.001
Thrombosed false lumen	0.47	0.21-0.95	.047	0.48	0.21-0.97	.054
Age, per y	1.01	0.99-1.04	.23	1.01	0.99-1.03	.26
Total arch replacement	1.71	0.97-3.02	.06	1.73	0.98-3.05	.059
Femoral artery perfusion	1.07	0.55-2.23	.85	1.06	0.55-2.21	.87
Redo surgery	–	–	–	1.84	0.40-6.04	.36

### Risk Factors for New Ischemic Stroke and 30-Day Mortality

Logistic regression analysis of the all cohort (total 85 stroke events [6.1%]) revealed that thrombosed false lumen was not an independent risk factor for new-onset ischemic stroke (OR, 1.04; 95% CI, 0.61-1.73; *P* = .88) (Table 5). The only significant independent predictor of ischemic stroke was salvage surgery (OR, 4.57; 95% CI, 1.94-10.6; *P* < .001). For 30-day mortality analysis, 75 deaths (5.3%) occurred within 30 days. The primary multivariate analysis confirmed 4 independent predictors: malperfusion (OR, 3.77; 95% CI, 2.22-6.39; *P* < .001), salvage surgery (OR, 5.36; 95% CI, 2.50-11.6; *P* < .001), shock (OR, 2.71; 95% CI, 1.45-4.93; *P* = .001), and thrombosed false lumen (OR, 0.47; 95% CI, 0.21-0.95; *P* = .047) (Table 4). In the multivariable regression sensitivity analysis, including redo surgery as an additional covariate, thrombosed false lumen showed a consistent protective trend but with attenuated statistical significance (OR, 0.48; 95% CI, 0.21-0.97; *P* = .054) (Table 4).

**Table 5:** Risk factors for ischemic stroke: Logistic regression analysis

Variable	Univariable analysis			Multivariable analysis		
	Odds ratio	95% CI	*P* value	Odds ratio	95% CI	*P* value
Salvage surgery∗	5.09	2.47-9.83	<.001	4.57	1.94-10.59	<.001
Shock	1.85	1.11-2.99	.02	1.25	0.65-2.26	.48
Age, per y	0.99	0.98-1.01	.24	0.99	0.97-1.01	.23
Femoral artery perfusion	1.06	0.61-1.99	.84	1.12	0.64-2.13	.70
Thrombosed false lumen	0.93	0.56-1.50	.77	1.04	0.61-1.73	.88
Entry at ascending aorta	0.82	0.53-1.28	.37	0.82	0.51-1.31	.40
Ascending localized (DeBakey type II)	0.84	0.43-1.51	.58	0.91	0.44-1.74	.79

∗ Salvage surgery is defined as emergency surgery performed in patients with cardiac arrest or extreme hemodynamic instability requiring cardiopulmonary resuscitation and/or mechanical circulatory support.

## Discussion

**Figure 2:**
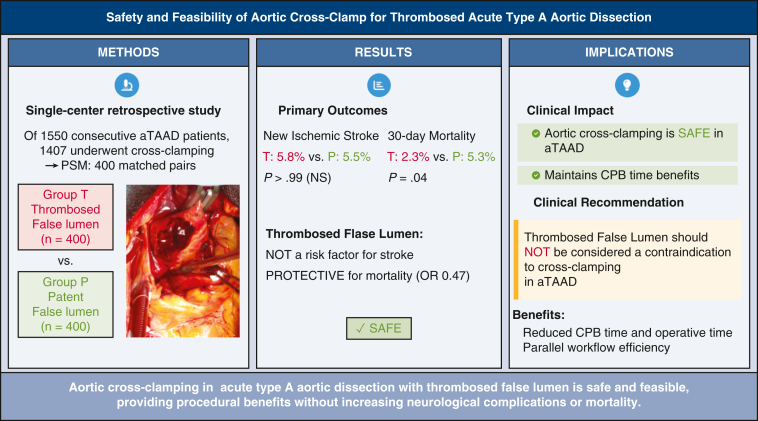
Graphical abstract summarizing the study methodology, primary outcomes, and clinical implications. The intraoperative photograph shows crossclamp application to the ascending aorta with thrombosed false lumen. *aTAAD*, Acute type A aortic dissection; *PSM*, propensity score matching; *NS*, not significant; *OR*, odds ratio; *CPB*, cardiopulmonary bypass.

In this large, single-center retrospective study of 1407 patients with aTAAD who underwent aortic crossclamping, we found that thrombosed false lumen was not associated with increased risk of new ischemic stroke or 30-day mortality compared with patent false lumen (Figure 2). These findings address the theoretical concern of increased thromboembolism risk associated with crossclamping a thrombosed dissected aorta and provide evidence supporting the feasibility of this surgical approach.

Our findings are consistent with previous studies demonstrating the safety of aortic crossclamping in aTAAD surgery. We previously reported on our systematic approach to aTAAD with aortic crossclamping, showing no association with operative mortality (adjusted OR, 0.77; 95% CI, 0.32-2.05; *P* = .58) or postoperative stroke (adjusted OR, 0.75; 95% CI, 0.3-1.92; *P* = .542) in 702 consecutive patients.^13^ Similarly, Immer and colleagues^14^ found no significant differences in in-hospital mortality (15.2% vs 17.6%; *P* = .633) or neurologic complications (9.4% vs 10.8%; *P* = .734) between crossclamped and nonclamped groups. However, these studies did not specifically examine false lumen thrombosis status. Our study extends this evidence by demonstrating safety specifically in the high-risk subgroup of patients with thrombosed false lumen, where theoretical embolic concerns have traditionally limited crossclamping.

The protective effect of thrombosed false lumen for 30-day mortality in our primary analysis is consistent with recent evidence. A meta-analysis by Zhang and colleagues^15^ and a study by Sakamoto and colleagues^16^ demonstrated higher mortality rates and elevated D-dimer levels in patent false lumen cases. In patent false lumen, continuous blood flow maintains perfusion pressure, increasing wall tension and the risks of expansion, redissection, and rupture.^17^ This ongoing flow predisposes patients to extensive dissection with organ malperfusion^18^ and coagulopathy that prolongs operative times.^16^ In contrast, thrombosed false lumen reduces intraluminal pressure and wall tension, promoting hemodynamic stabilization while potentially sealing sites of dissection extension.^19^ Although our sensitivity analyses suggest caution in attributing the observed mortality benefit solely to thrombosis status because it may also reflect less severe disease presentations requiring fewer salvage interventions and redo surgeries, the biological plausibility and consistent direction of effect across all analyses support a potential protective role of false lumen thrombosis in aTAAD surgery. The primary theoretical concern with crossclamping in thrombosed false lumen aTAAD is potential thrombus dislodgement causing cerebral embolism. However, our findings do not support this risk because ischemic stroke rates were similar between groups in primary analysis, propensity score-matching sensitivity analysis, and multivariable regression analysis. This complete consistency of stroke outcomes across all analytical approaches strongly supports the safety of crossclamping in thrombosed false lumen. Several factors may explain this safety profile. When the intimal entry is distant from the clamping site, thrombus displacement through remote entry tears is unlikely. Even with proximal entries, complete thrombosis may maintain true lumen pressure stability, preventing thrombus mobilization. In this study, logistic regression analysis suggested that thrombosed type, entry at ascending aorta, and extension of dissection was not risk factor for stroke. No morphological characteristics of aortic dissection could be identified as risk factors for embolism. Otherwise, our multivariate analysis identified salvage surgery as the strongest predictor of ischemic stroke, consistent with previous reports highlighting the importance of early intervention before hemodynamic collapse.^20^^,^^21^ Previous studies have identified malperfusion syndrome, age, and circulatory arrest duration as main neurological predictors, whereas crossclamping itself was not a significant factor.^14^

Based on our high success rate (99.8%) and experience with 1407 cases, we recommend that thrombosed false lumen should not be considered an absolute contraindication to crossclamping. Moreover, crossclamping offers significant procedural advantages that may outweigh theoretical embolic risks. These benefits include reduced CPB time and operative time through the ability to perform proximal procedures during systemic cooling, decreased risk of postoperative renal dysfunction,^22^^,^^23^ and prevention of left ventricular distention in cases with severe aortic regurgitation. However, individualized decision making remains essential. First, in cases utilizing apical cannulation where the cannula is positioned in the ascending aorta, crossclamping becomes technically unfeasible. Second, in cases where beating coronary artery bypass grafting is performed first or in redo cases, crossclamping is unnecessary because cooling is completed during bypass grafting and adhesiolysis. Furthermore, it should be noted that this study exclusively examined acute dissections. In chronic phase dissections, thrombus can be firm and the entry may be widely patent, potentially serving as an embolic source; therefore, we avoid crossclamping in these cases at our institution. Finally, in cases with significant ascending aortic dilatation (>50-60 mm), we avoid crossclamping due to rupture risk and potential for incomplete clamping.

For successful crossclamping, our technical recommendation involves adequate dissection of the posterior aortic aspect using a widely curved clamp, as demonstrated in Video 1. This approach facilitates safe aortic resection and proximal manipulation. Following crossclamp application, it is crucial to avoid abrupt aortic incision because this may dislodge fresh thrombus into the left ventricle or coronary ostium, causing an embolism. Instead, we recommend initially incising only the adventitial layer, allowing for careful removal of thrombus from the false lumen before proceeding with complete aortic transection. All crossclamped aortic tissue must be completely resected during distal anastomosis due to its potential compromise. Successful crossclamping facilitates proximal procedures during the cooling phase of CPB, allowing for a parallel workflow, although the clinical significance of the approximately 20-minute time savings was not clarified in this study; further study is required.

Given our experience with cases requiring declamping, we implement continuous monitoring using regional cerebral oxygen saturation at 6 sites—bilateral forehead, both upper limbs, and both lower limbs—to enable early detection and intervention. We have previously reported on intraoperative newly developed malperfusion.^24^ This phenomenon occurs when altered hemodynamics following cardiopulmonary bypass initiation increases blood flow to the false lumen, resulting in compression and obstruction of the true lumen. Although typically observed at the onset of CPB, the 2 cases we experienced occurred during aortic crossclamping. Furthermore, both cases involved patent false lumen dissections with femoral artery perfusion, suggesting that vigilant monitoring throughout the entire surgical procedure is essential, particularly in this patient subtype.

### Limitations

Several limitations should be acknowledged. The retrospective design introduces potential selection and information bias. Although propensity score matching balanced observed covariates, residual imbalances remained in the primary analysis for salvage surgery, redo surgery, Marfan syndrome, and partial arch replacement. To address these concerns, we performed comprehensive sensitivity analyses. Enhanced propensity score matching, including salvage surgery and redo surgery as matching variables, confirmed consistent safety findings for stroke and showed similar trends for mortality. Additionally, multivariable regression sensitivity analysis, including redo surgery as a covariate, demonstrated comparable attenuation of the protective effect of the thrombosed type. These results suggests that the apparent protective effect of thrombosed false lumen may be partially confounded by differential frequency of extreme clinical presentations and surgical complexity, particularly salvage surgery and redo procedures. Surgeon preference in crossclamping decisions or perfusion site selection may introduce selection bias. Stroke detection relied on clinical assessment and indicated imaging studies, potentially underestimating true cerebral embolism incidence. Because propensity score matching analyzes only a subset of patients, our findings may not be fully generalizable to the entire cohort. Finally, potential embolic risks from thrombosed false lumen crossclamping might be offset by inherent cerebral infarction risks in patent false lumen cases, potentially masking true differences. These limitations warrant further investigation with larger multicenter cohorts and longer follow-up.

## Conclusions

In this large, propensity score-matched analysis, aortic crossclamping in patients with aTAAD with thrombosed false lumen was not associated with an increased risk of ischemic stroke or early mortality compared with patients with patent false lumen. This suggests that aortic crossclamping is a safe and feasible technique in aTAAD surgery regardless of false lumen status.

## Conflict of Interest Statement

The authors reported no conflicts of interest.

The *Journal* policy requires editors and reviewers to disclose conflicts of interest and to decline handling or reviewing manuscripts for which they may have a conflict of interest. The editors and reviewers of this article have no conflicts of interest.

## Figures and Tables

**Table E1:** Baseline characteristics in sensitivity analysis with enhanced propensity score matching∗

Variable	Group T (n = 399)	Group P (n = 399)	SMD
Age (y)	73 (65-80)	72 (65-80)	0.002
Female sex	224 (56.1)	231 (57.9)	0.035
Body surface area (m^2^)	1.58 (1.44-1.78)	1.60 (1.47-1.74)	0.002
Hypertension	300 (75.2)	295 (73.9)	0.029
Dyslipidemia	96 (24.1)	98 (24.6)	0.012
Diabetes mellitus	23 (5.8)	26 (6.5)	0.031
Creatinine >2 mg/dL	17 (4.3)	12 (3.0)	0.067
Marfan syndrome	5 (1.3)	2 (0.5)	0.081
Malperfusion	34 (8.5)	33 (8.3)	0.009
Shock	75 (18.8)	78 (19.5)	0.019
Neurological symptoms	20 (5.0)	20 (5.0)	<0.001
Salvage surgery	11 (2.8)	10 (2.5)	0.016
Redo surgery	1 (0.3)	4 (1.0)	0.095
Femoral artery perfusion	328 (82.2)	314 (78.7)	0.089
Total arch replacement	66 (16.5)	70 (17.5)	0.027
Root surgery	19 (4.8)	18 (4.5)	0.012

Data are presented as n (%) for categorical variables and median (interquartile range) for continuous variables. *Group T*, Thrombosed false lumen; *Group P*, patent false lumen; *SMD*, standardized mean difference.

∗ Enhanced propensity score matching included salvage surgery and redo surgery as additional matching variables.

**Table E2:** Outcomes in sensitivity propensity score-matching analysis∗

Outcome	Group T (n = 399)	Group P (n = 399)	*P* value
Operative time			
Cardiopulmonary bypass time (min)	124 (107-150)	124 (106-151)	.95
Aortic crossclamp time (min)	88 (73-106)	87 (72-105)	.79
Circulatory arrest time (min)	27 (22-33)	26 (21-32)	.50
Operation time (min)	218 (185-268)	224 (190-275)	.24
Early outcomes			
New ischemic stroke	23 (5.8)	20 (5.1)	.64
Spinal cord injury	5 (1.3)	5 (1.3)	1.00
Acute kidney injury†	31 (7.8)	45 (11.3)	.09
Requirement for renal replacement therapy	9 (2.3)	14 (3.5)	.30
Respiratory failure‡	28 (7.1)	33 (8.3)	.59
30-d mortality	9 (2.3)	17 (4.3)	.12

Values are presented as n (%) for categorical variables and median (interquartile range) for continuous variables. *Group T*, Thrombosed false lumen; *Group P*, patent false lumen.

∗ Sensitivity propensity score matching included salvage surgery and redo surgery as additional matching variables in addition to all variables used in the primary analysis.

† Acute kidney injury is defined as Kidney Disease Improving Global Outcomes stage 2 or greater.

‡ Respiratory failure is defined as prolonged ventilation >72 hours or requirement for tracheotomy.

## Supplementary Data

Video 1 Surgical technique of aortic crossclamp for thrombosed acute type A aortic dissection. Video available at: https://www.jtcvs.org/article/S2666-2736(25)00397-3/fulltext.
