# Supplementary figures and images for: Safety and feasibility of aortic crossclamp for thrombosed acute type A aortic dissection
Source: JTCVS Open. 2025 Nov 10;29:101505. doi: 10.1016/j.xjon.2025.10.026 (PMC13059968; doi:10.1016/j.xjon.2025.10.026)

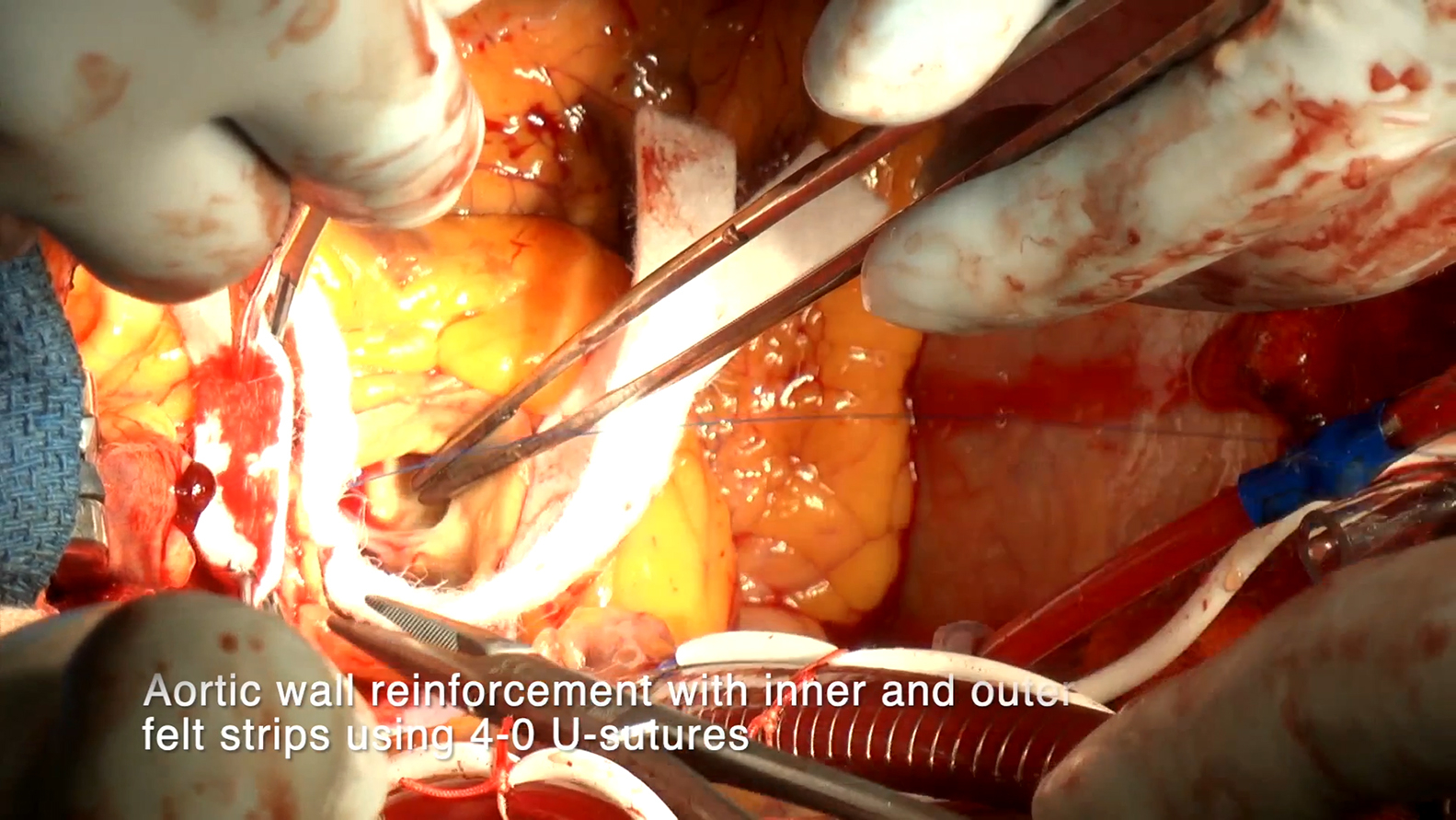

Supplement: Video 1 — Surgical technique of aortic crossclamp for thrombosed acute type A aortic dissection. Video available at: https://www.jtcvs.org/article/S2666-2736(25)00397-3/fulltext. [file fx2.jpg]
